# Recreational water illness in Canada: a changing risk landscape in the context of climate change

**DOI:** 10.17269/s41997-022-00688-8

**Published:** 2022-09-16

**Authors:** Ian Young, J. Johanna Sanchez, Jordan Tustin

**Affiliations:** School of Occupational and Public Health, Toronto Metropolitan University, 350 Victoria Street, Toronto, Ontario M5B 2K3 Canada

**Keywords:** Water pollution, Water quality, Waterborne diseases, Bathing beaches, Climate change, Pollution de l’eau, qualité de l’eau des plages, maladies hydriques, plage pour la baignade, changement climatique

## Abstract

Swimming and other recreational water activities at public beaches are popular outdoor leisure activities among Canadians. However, these activities can lead to increased risks of acquiring acute gastrointestinal illness and other illnesses among beachgoers. Young children have much higher rates of exposure and illness than other age groups. These illnesses have a significant health and economic burden on society. Climate change is expected to influence both the risk of exposure and illness. A warming climate in Canada, including more severe summer heatwave events, will likely lead to increased recreational water use. Warmer temperatures will also contribute to the growth and increased range of harmful algal blooms and other climate-sensitive pathogens. Increased precipitation and heavy rainfall events will contribute to fecal and nutrient contamination of beach waters, increasing risks of gastrointestinal illness and harmful algal bloom events. There is a need to enhance recreational water research and surveillance in Canada to prepare for and adapt to these changing risks. Key research and policy needs are suggested and discussed, including evaluating and monitoring risks of recreational water illness in Canadian contexts, improving timely reporting of recreational water quality conditions, and enhancing approaches for routine beach water surveillance.

## Résumé

La natation et les autres activités nautiques récréatives sur les plages publiques sont des activités de loisirs en plein air populaires chez les Canadiens. Cependant, ces activités peuvent entraîner des risques accrus de contracter des maladies gastro-intestinales aiguës et d’autres maladies chez les baigneurs. Les jeunes enfants ont des taux d’exposition et d’infection beaucoup plus élevés que les autres groupes d’âge. Ces maladies représentent un lourd fardeau sanitaire et économique pour la société. On s’attend à ce que le changement climatique influence à la fois le risque d’exposition et d’infection. Un réchauffement climatique au Canada, y compris des vagues de chaleur estivales plus intenses, entraînera probablement une utilisation accrue de l’eau à des fins récréatives. Des températures plus chaudes contribueront également à la prolifération d’algues nuisibles à la santé et la croissance d’autres agents pathogènes sensibles au climat. L’augmentation des précipitations et des pluies abondantes contribueront à la contamination fécale et nutritive des eaux des plages, augmentant les risques de maladies gastro-intestinales et d’efflorescences algales nocives. Il est nécessaire d’améliorer la recherche et la surveillance des eaux récréatives au Canada pour se préparer et s’adapter à ces risques changeants. Les principaux besoins en matière de recherche et de politiques sont suggérés et discutés, y compris l’évaluation et la surveillance des risques de maladie des eaux récréatives dans les contextes canadiens, l’amélioration des rapports en temps opportun sur les conditions de qualité des eaux récréatives et l’amélioration des approches de surveillance de routine des eaux de plage.

## Introduction

Swimming and other recreational water activities (e.g., water sports, kayaking) at public beaches are increasingly popular summertime activities in Canada. For example, a national survey in 2015 found that nearly 30% of Canadians reported engaging in any recreational water activities in the prior 7-day period in the summer (Janicki et al., 2018). Children <10 years of age were more than four times as likely as adults to engage in these activities (Janicki et al., 2018). Recreational water activities will likely become more popular in the future as Canadians cope with the impacts of climate change. Specifically, higher ambient air temperatures and more severe heatwave events are expected in Canada (Bush & Lemmen, 2019), which will likely increase beach use and recreational water exposures.

## Public health burden of recreational water illness

Globally, there is strong evidence that swimming and other recreational water activities with a high likelihood of water immersion (e.g., surfing, snorkeling) are associated with an elevated risk of various illnesses, particularly acute gastrointestinal illness (AGI), among beachgoers (Leonard et al., 2018; Russo et al., 2020). Children and youth are disproportionally affected by AGI, as they tend to spend more time in the water, swallow more water when swimming, and have developing immune and digestive systems (Arnold et al., 2016; Deflorio-Barker et al., 2018a). For example, previous cohort studies in the United States (US) have found that the risk of AGI due to swimming among children aged 0–4 years was more than double that of those aged 10+ years (22.4 vs. 7.8 episodes per 1000 beachgoers) (Arnold et al., 2016). There are also sex- and gender-related differences in recreational water exposures, as male children tend to spend more time in the water and swallow more water when swimming than females (Deflorio-Barker et al., 2018a).

Recreational water illness (RWI) is under-reported and under-diagnosed in Canada, as those with self-limiting illness may not seek medical attention or submit samples for diagnosis, and illnesses may be misattributed to food-borne exposures. These illnesses have a significant burden and cost to society. Although we lack RWI burden data in Canada, approximately 90 million cases are estimated to occur in the US each year, costing US$2.2–3.7 billion annually (DeFlorio-Barker et al., 2018b).

## Fecal contamination, AGI, and climate change

The risk of AGI from recreational water contact is caused by exposure to various enteric pathogens that contaminate beach water from human and animal fecal sources. Enteric viruses (e.g., norovirus) are frequently associated with human sewage contamination and are the most common infectious agents responsible for RWI (Soller et al., 2010). Human sewage contamination at beaches can occur from treated wastewater effluent and, more importantly, via untreated sewage from combined sewer overflow events (Soller et al., 2010). Combined sewer overflows occur in municipalities with older, combined sewage and stormwater systems. Heavy rainfall events can overwhelm the capacity of such systems, resulting in the release of untreated human sewage directly into nearby water bodies. In agricultural areas, rainfall events can also lead to contamination of beach waters with animal manure, also increasing AGI risks among beachgoers. Climate change will result in a greater frequency and intensity of heavy rainfall events in many parts of Canada (Bush & Lemmen, 2019), which will contribute to increased beach water fecal contamination.

## Harmful algal blooms and other infectious disease agents of concern

Harmful algal blooms (HABs) in recreational freshwater bodies are commonly reported in Canada due to the growth of photosynthetic cyanobacteria. HABs due to cyanobacteria are a public health concern because they can produce various cyanotoxins and contain cell-surface endotoxins that can lead to human exposure through water ingestion, inhalation of aerosols, or skin contact (Carmichael & Boyer, 2016; Health Canada, 2022). Exposure to these toxins can cause a variety of acute symptoms (e.g., AGI, generalized illness, skin irritations) as well as more severe hepatotoxic and neurologic effects (Carmichael & Boyer, 2016; Roberts et al., 2020). For example, a 2009 study at three Quebec lakes found that various recreational water activities were associated with increased risks of AGI among lake users (Lévesque et al., 2014). Additionally, HABs are a One Health issue as they also negatively affect animal and ecosystem health (Roberts et al., 2020). HABs are most often caused by eutrophication of water bodies, especially due to increases in phosphorus, and their growth is promoted through high water temperatures (Carmichael & Boyer, 2016). Therefore, climate change will likely contribute to the proliferation of HABs via its impact on warming water temperatures and increasing precipitation and heavy rainfall events which can affect nutrient loadings in water bodies.

Another climate-sensitive pathogen of concern for beach water exposures is *Naegleria fowleri*, a free-living aquatic amoeba that causes primary amoebic meningoencephalitis (PAM). PAM is a rare but usually fatal brain infection that results from recreational water exposure to the amoeba. An analysis of cases in the US from 1978 to 2018 found a northward expansion of PAM cases and an association with higher ambient air temperatures (Gharpure et al., 2021). Canada should anticipate possible recreational water exposures to this pathogen in the future due to a likely increase in range of the amoeba due to warming temperatures. Climate change may also increase the prevalence of “swimmer’s itch” (i.e., cercarial dermatitis), which is an allergic rash that develops from exposure to avian schistosomes in natural water bodies, as warmer temperatures and eutrophication can both lead to increased schistosome development (Gordy et al., 2018).

## Recreational water quality surveillance in Canada

Health Canada has developed national recreational water quality guidelines to assist local and provincial health and environmental authorities with their recreational water surveillance programs (Health Canada, 2021). The guidelines recommend that authorities routinely monitor public beaches for indicators of fecal contamination (*Escherichia coli* and/or enterococci) and HABs (Health Canada, 2021, 2022). For fecal indicators, “beach action values” have been established corresponding to acceptable levels of AGI risks from prior US cohort studies (Health Canada, 2021). If these values are exceeded in any beach water samples, public health actions (e.g., swimming advisories) are recommended. The specific approaches to beach monitoring are established at the provincial or local level depending on the Canadian jurisdiction, and these practices can vary widely. For example, in Ontario, each of the 34 public health units has different frequencies of beach monitoring, ranging from daily to monthly depending on beach popularity, historical conditions, resources, and other factors (Heasley et al., 2022).

## Key research and policy needs in Canada

The current Health Canada “beach action values” are based on RWI data from the US. The last RWI cohort study to be conducted in Canada was in 1980 (Seyfried et al., 1985), and there is an urgent need for additional cohort studies to examine the burden and risk of RWI in a modern Canadian context. Additionally, quantitative microbial risk assessments are needed to evaluate RWI risks under different local and site-specific conditions and settings. At the national level, a systematic surveillance system for RWI outbreaks and HAB events is needed, ideally using a One Health approach, to facilitate public health interventions and trend analysis.

Current beach water surveillance approaches in Canada rely on culture-based testing for fecal indicators. There is a 24–48-h delay in receiving results from these tests. This affects timely and accurate public health actions, as beach advisory notifications are made based on previous day conditions. The most recent Health Canada guidelines recommend that authorities explore the use of rapid testing methods (quantitative PCR) for enterococci in beach water (Health Canada, 2021). These methods, which can return results in only 3–4 h, have been successfully adopted in some US settings (e.g., Chicago) and should be evaluated for implementation in Canadian jurisdictions (Shrestha & Dorevitch, 2020). Additionally, real-time predictive models can be developed to determine beach water quality results based on environmental and weather conditions. The accuracy of these methods varies widely depending on the context and methodology used (Heasley et al., 2021). Promising methods such as artificial neural networks and Bayesian networks warrant further investigation in Canada to improve beach notifications.

Numerous enhancements can be made to beach water surveillance practices at the local and provincial levels. Microbial source tracking methods can be used at beaches with frequently high levels of fecal contamination to determine the relative contribution of different pollution sources (e.g., human, cattle, wildlife). This information can be used to target pollution prevention efforts (Edge et al., 2018). Routine water surveillance should also include consistent and accurate measurement of important environmental predictors of beach water quality (e.g., water turbidity, heavy rainfall events). Such information can support the development of predictive models and guide risk communication messaging. Finally, authorities should investigate the role of citizen science approaches to enhance beach water surveillance, including public reporting of indicators of poor water quality (e.g., high turbidity, visible HABs). Climate change will affect recreational water quality and beach exposures in Canada, increasing RWI risks, and there is a critical need for enhanced research, surveillance, and adaptation strategies to mitigate these risks.

## References

[CR1] Arnold BF, Wade TJ, Benjamin-Chung J, Schiff KC, Griffith JF, Dufour AP (2016). Acute gastroenteritis and recreational water: Highest burden among young US children. American Journal of Public Health.

[CR2] Bush, E., & Lemmen, D. (2019). *Canada’s changing climate report*. Ottawa. Available at: https://www.nrcan.gc.ca/sites/www.nrcan.gc.ca/files/energy/Climate-change/pdf/CCCR_FULLREPORT-EN-FINAL.pdf. Accessed 2 May 2022.

[CR3] Carmichael WW, Boyer GL (2016). Health impacts from cyanobacteria harmful algae blooms: implications for the North American Great Lakes. Harmful Algae.

[CR4] Deflorio-Barker S, Arnold BF, Sams EA, Dufour AP, Colford JM, Weisberg SB (2018). Child environmental exposures to water and sand at the beach: Findings from studies of over 68,000 subjects at 12 beaches. Journal of Exposure Science & Environmental Epidemiology.

[CR5] DeFlorio-Barker S, Wing C, Jones RM, Dorevitch S (2018). Estimate of incidence and cost of recreational waterborne illness on United States surface waters. Environmental Health.

[CR6] Edge TA, Hill S, Crowe A, Marsalek J, Seto P, Snodgrass B (2018). Remediation of a beneficial use impairment at Bluffer’s Park beach in the Toronto area of concern. Aquatic Ecosystem Health & Management.

[CR7] Gharpure R, Gleason M, Salah Z, Blackstock AJ, Hess-Homeier D, Yoder JS (2021). Geographic range of recreational water-associated primary amebic meningoencephalitis, United States, 1978-2018. Emerging Infectious Diseases.

[CR8] Gordy MA, Cobb TP, Hanington PC (2018). Swimmer’s itch in Canada: A look at the past and a survey of the present to plan for the future. Environmental Health.

[CR9] Health Canada. (2021). *Guidelines for Canadian recreational water quality: Indicators of fecal contamination*. Available at: https://www.canada.ca/en/health-canada/programs/consultation-guidelines-recreational-water-quality-fecal-contamination/document.html. Accessed 2 May 2022.

[CR10] Health Canada. (2022). *Guidelines for Canadian recreational water quality – Cyanobacteria and their toxins*. Available at: https://www.canada.ca/en/health-canada/services/publications/healthy-living/guidance-canadian-recreational-water-quality-cyanobacteria-toxins.html. Accessed 2 May 2022.

[CR11] Heasley C, Sanchez J, Young I, Tustin J (2022). Beach water monitoring practices and challenges in Ontario public health units. Environmental Health Review.

[CR12] Heasley C, Sanchez JJ, Tustin J, Young I (2021). Systematic review of predictive models of microbial water quality at freshwater recreational beaches. PLoS ONE.

[CR13] Janicki R, Thomas KM, Pintar K, Fleury M, Nesbitt A (2018). Drinking and recreational water exposures among Canadians: Foodbook study 2014–2015. Journal of Water and Health.

[CR14] Leonard AF, Singer A, Ukoumunne OC, Gaze WH, Garside R, AFC L (2018). Is it safe to go back into the water? A systematic review and meta-analysis of the risk of acquiring infections from recreational exposure to seawater. International Journal of Epidemiology.

[CR15] Lévesque B, Gervais MC, Chevalier P, Gauvin D, Anassour-Laouan-Sidi E, Gingras S (2014). Prospective study of acute health effects in relation to exposure to cyanobacteria. Science of the Total Environment.

[CR16] Roberts VA, Vigar M, Backer L, Veytsel GE, Hilborn ED, Hamelin EI (2020). Surveillance for harmful algal bloom events and associated human and animal illnesses — One health harmful algal bloom system, United States, 2016–2018. Morbidity and Mortality Weekly Report.

[CR17] Russo GS, Eftim SE, Goldstone AE, Dufour AP, Nappier SP, Wade TJ (2020). Evaluating health risks associated with exposure to ambient surface waters during recreational activities: A systematic review and meta-analysis. Water Research.

[CR18] Seyfried PL, Tobin RS, Brown NE, Ness PF (1985). A prospective study of swimming-related illness I. Swimming-associated health risk. American Journal of Public Health.

[CR19] Shrestha A, Dorevitch S (2020). Slow adoption of rapid testing: Beach monitoring and notification using qPCR. Journal of Microbiological Methods.

[CR20] Soller JA, Schoen ME, Bartrand T, Ravenscroft JE, Ashbolt NJ (2010). Estimated human health risks from exposure to recreational waters impacted by human and non-human sources of faecal contamination. Water Research.

